# Transabdominal Preperitoneal Repair for an External Supravesical Hernia With an Incarcerated Ovary: A Case Report

**DOI:** 10.7759/cureus.60111

**Published:** 2024-05-11

**Authors:** Goshi Fujimoto, Takashi Deguchi, Junya Shirai

**Affiliations:** 1 Gastroenterological Surgery, Koga Community Hospital, Yaizu, JPN

**Keywords:** ovary, hernia, suprapubic incisional hernia, inguinal hernia, femoral hernia, case report, incarcerated ovary, supravesical hernia, transabdominal preperitoneal (tapp)

## Abstract

External supravesical hernias with ovarian incarceration have not been reported previously. Here, we describe transabdominal preperitoneal (TAPP) repair of an external supravesical hernia with ovarian incarceration. A 68-year-old woman presented to our outpatient clinic with the chief complaint of right inguinal swelling and pain. A 3-cm-diameter mass in the right inguinal region that was difficult to reduce was palpable, and computed tomography (CT) revealed a suspicious lesion of the right hydrocele of the canal of Nuck. Hydrocelectomy was performed through an inguinal incision, and the external inguinal ring was repaired using the Marcy method. The histopathological examination confirmed the diagnosis of the canal of Nuck. Three months postoperatively, the patient again presented with right inguinal pain, and CT revealed a right femoral hernia requiring surgical repair. Intraoperative findings revealed a right external supravesical hernia with an incarcerated ovary, which was laparoscopically reduced and repaired with a mesh. At the three-month follow-up, there were no postoperative complications or recurrences. Incarcerated ovaries with inguinal hernias have been reported in girls; however, incarcerated ovaries with external supravesical hernias have not been reported in women. Although the preoperative diagnosis was difficult to make in this case, the laparoscopic approach led to the diagnosis and successful mesh repair. Although optimal mesh repair of external supravesical hernias using TAPP has not been established, we believe that 2-5 cm around the hernial orifice, the Hesselbach triangle, and the lateral triangle should be covered with mesh.

## Introduction

Supravesical hernia is a rare condition accounting for 3.4% of all inguinal hernias [1]. External supravesical hernias manifest primarily as direct inguinal hernias and are often misidentified as true inguinal hernias [2]. The diagnosis of external supravesical hernias is expected to increase as laparoscopic surgery becomes more common; however, optimal mesh repair by laparoscopic transabdominal preperitoneal (TAPP) repair has not yet been established [2]. The ovaries, small intestine, colon, and greater omentum may also be involved in protruding inguinal hernias. Although the ovary is included in 15-20% of female inguinal hernia cases [3], external supravesical hernias with ovarian incarceration have not been reported. Here, we describe TAPP repair of an external supravesical hernia with ovarian incarceration to present the optimal area of mesh repair.

## Case presentation

**Figure 1:**
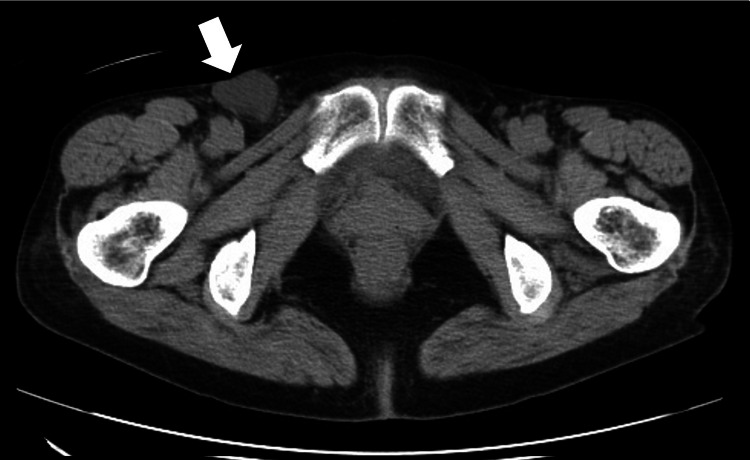
Preoperative plain abdominal CT scan Plain abdominal CT scan showing a suspicious lesion in the right hydrocele of the canal of Nuck (arrow) CT, computed tomography

A 68-year-old woman presented to our outpatient clinic with right inguinal distention for six months and right inguinal pain for one month. She had a history of hypertension and dyslipidemia and a body mass index of 18.5 kg/m^2^. A 3-cm-diameter mass in the right inguinal region that was difficult to reduce was noted, and a plain abdominal computed tomography (CT) scan showed a suspicious lesion in the right hydrocele of the canal of Nuck (Figure 1).

**Figure 2:**
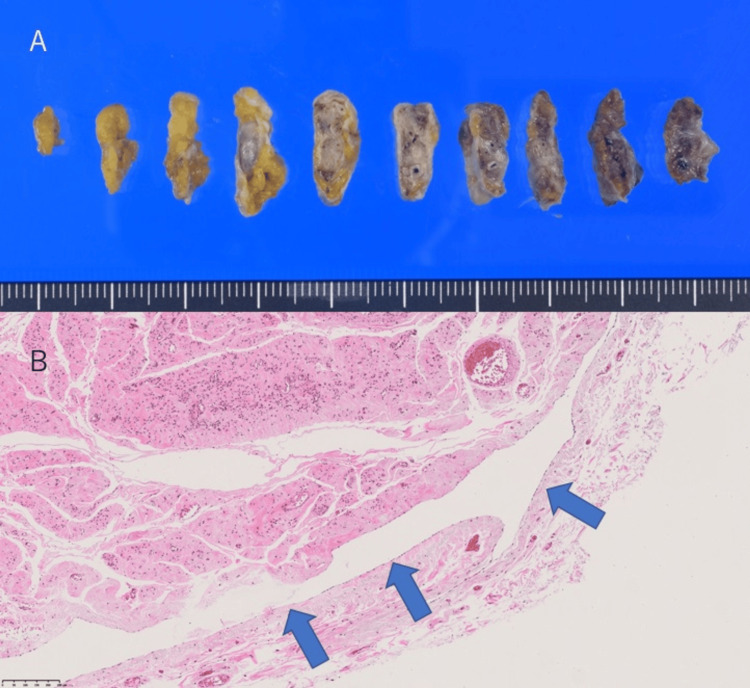
Histopathological examination Macroscopic findings of the resected specimen (A); Histopathological examination revealed a cyst covered by a single layer of mesothelium, a finding of the canal of Nuck (B)

The hydrocele was resected through an inguinal incision, and the external inguinal ring was repaired using the Marcy method. The histopathological examination revealed a hydrocele in the canal of Nuck (Figure 2).

**Figure 3:**
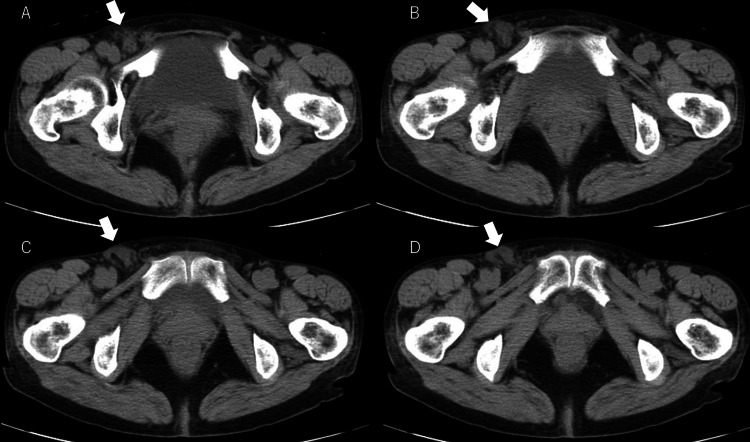
Plain abdominal CT scan after hydrocelectomy Plain abdominal CT scan revealing a right femoral hernia (arrows) CT, computed tomography

Three months postoperatively, the patient presented with bulging and pain in the right inguinal region. A bulge measuring 2-cm in diameter was observed in the right inguinal region. Plain abdominal CT revealed a right femoral hernia, so the patient underwent TAPP repair (Figure 3).

**Figure 4:**
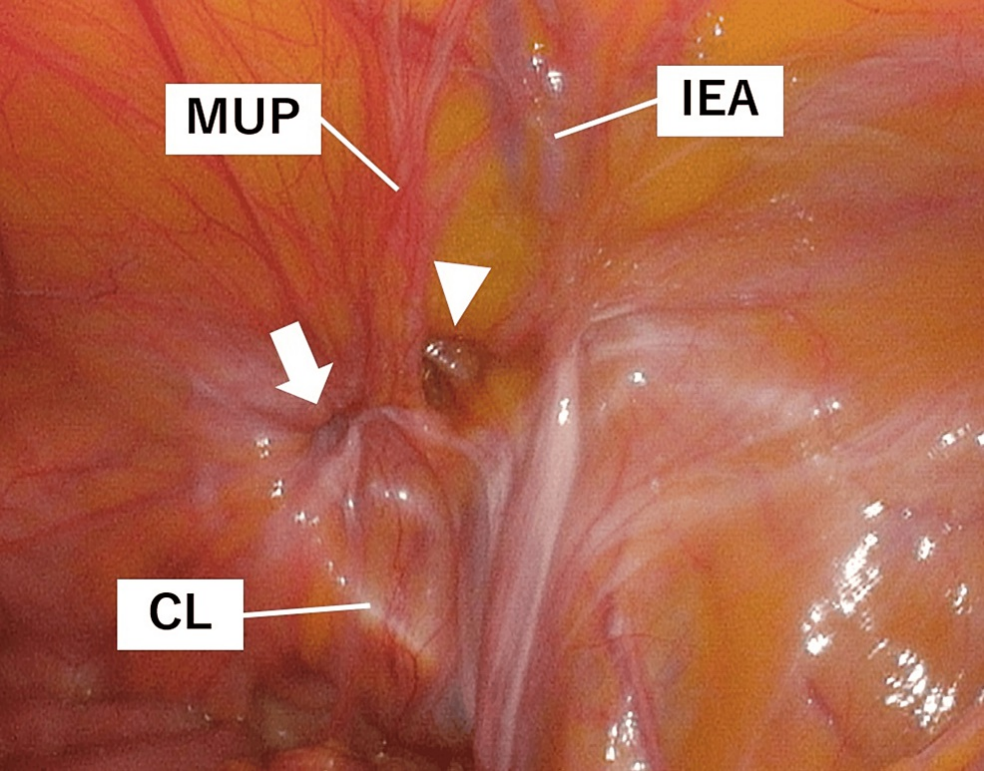
Laparoscopic findings of the external supravesical hernia Intraoperative findings revealing a right external supravesical hernia (arrow) with right ovarian incarceration and a right direct inguinal hernia (arrowhead) CL, Cooper ligament; IEA, inferior epigastric artery; MUP, medial umbilical plica

A 12-mm camera port was inserted into the patient’s umbilicus using the open method, and 5-mm ports were added to the right and left lateral abdomen. A 5-mm camera was used, and the patient was placed in a head-down position with an insufflation pressure of 10 mmHg. Intraoperative findings revealed a right external supravesical hernia with right ovarian incarceration, right direct inguinal hernia, left indirect inguinal hernia, and left femoral hernia (Figure 4).

**Figure 5:**
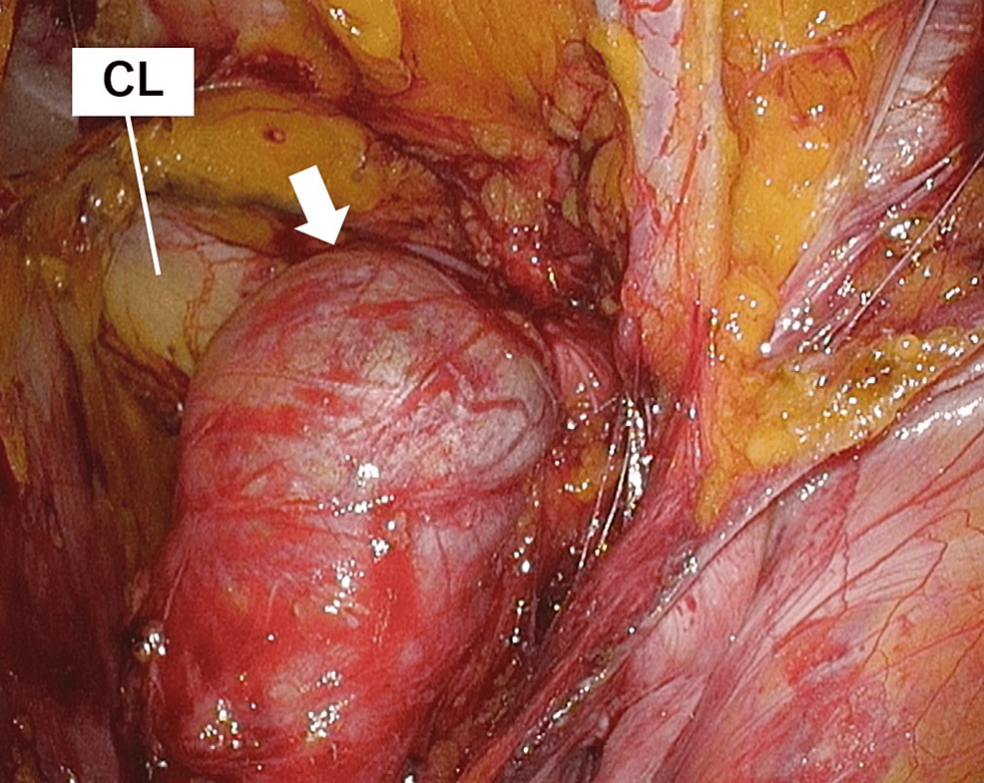
Inclusion of the right ovary in the femoral hernia The right ovary (arrow) is withdrawn into the abdominal cavity laparoscopically. CL, Cooper ligament

Additionally, the ovary was incarcerated in the form of a femoral hernia. The hepatic falciform ligament was partially defective, and the median umbilical fold was unclear. The ovary was withdrawn from the abdominal cavity and inverted through a peritoneal incision near the ovary (Figure 5).

**Figure 6:**
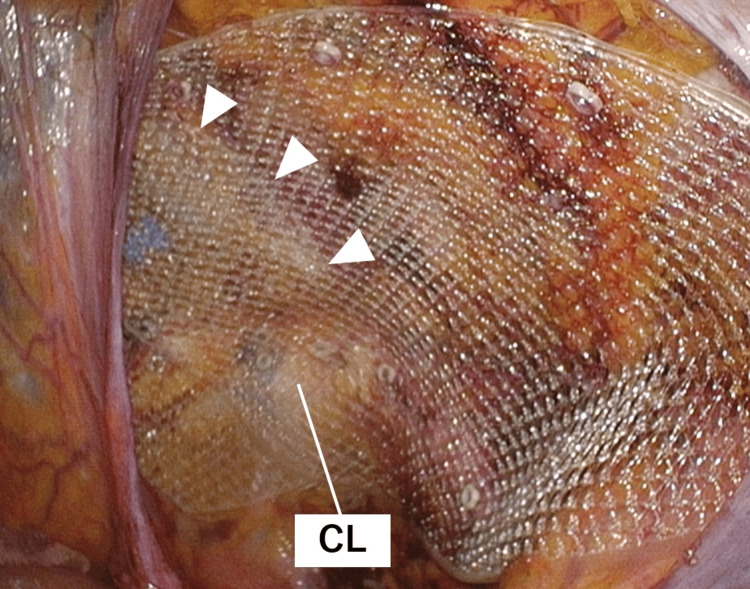
Bilateral mesh repair The hernial orifices on the right side are covered by a mesh overlapping the left side mesh (arrowheads) ventral to the pubic tubercle. CL, Cooper ligament

The right ovary showed no necrosis and thus was spared. Both hernias were repaired with 3D Max Light Mesh (L size, Bard) overlapping each other ventral to the pubic tubercle and fixed using CapSure (Bard) (Figure 6).

At the three-month follow-up, the patient had no postoperative complications or recurrences.

## Discussion

The supravesical fossa is located medial to the medial umbilical plica, lateral to the urachus, and on the cranial side of the iliopubic tract, pubis, and bladder [4]. In cases of supravesical hernias, the hernia orifice is found on the supravesical fossa; these hernias are classified as internal supravesical and external supravesical hernias. External supravesical hernias can appear as direct inguinal, femoral, obturator, or interparietal hernias depending on the direction of the protrusion [2]. Since a supravesical hernia is located medial to the Hesselbach triangle, surgeons often misidentify it as a true inguinal hernia [2]. Observation of the hernial orifice using TAPP repair may increase the diagnosis of external supravesical hernias.

Supravesical hernias develop owing to the failure of transversus abdominis aponeurosis and transversalis fascia integrity, and their pathogenesis is similar to that of direct inguinal hernias [1]. The coexistence of direct inguinal and external supravesical hernias has also been reported [5]. Therefore, the mesh should cover an optimal area for a direct inguinal hernia. The Hesselbach triangle is the most common site of recurrence after direct inguinal hernia repair. Recurrence in the form of indirect inguinal hernias is also observed following any of the procedures (mesh, laparoscopic, and Shouldice repair) for direct inguinal hernias [6]. Therefore, the mesh covers the medial side of the Hesselbach triangle and the lateral triangle, as in the usual direct inguinal hernia repair procedure. Additionally, because the patient in this case had a femoral hernia-type prolapse, the area around the femoral ring had to be securely covered with mesh.

**Figure 7:**
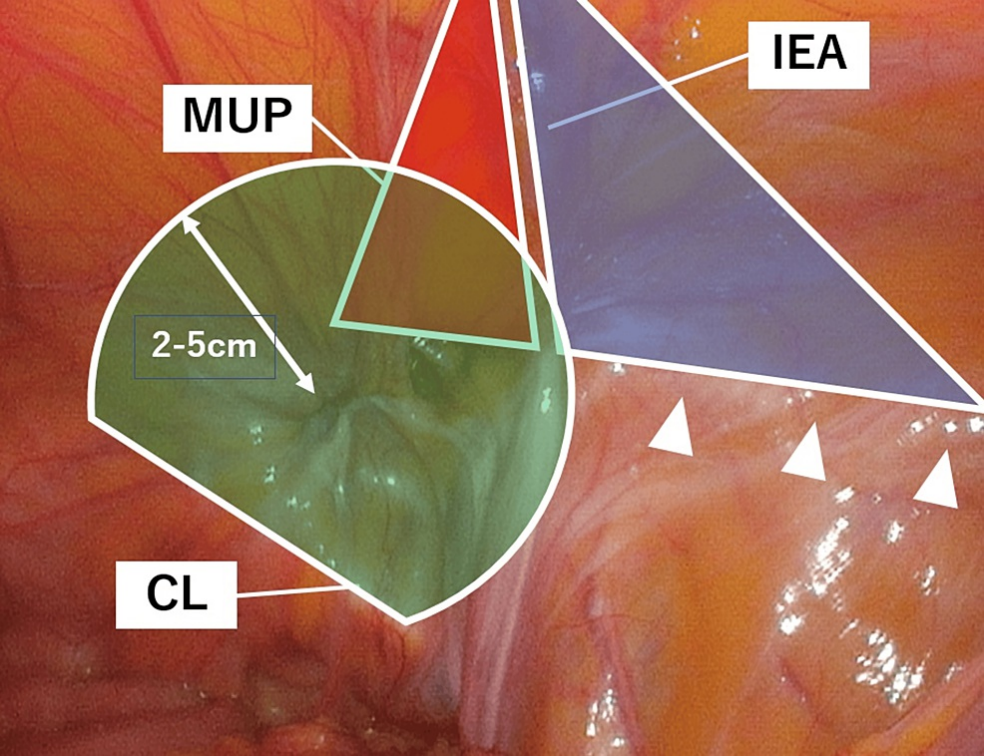
Optical area for mesh covering The mesh should cover an area of 2–5 cm around the hernial orifice (green area), Hesselbach triangle (red area), and lateral triangle (blue area) for TAPP repair of an external supravesical hernia. The Hesselbach triangle is the area bounded by the inferior epigastric vessels, the lateral edge of the abdominal rectal muscle, and the inguinal ligament (originally, they included the areas of the femoral ring). The lateral triangle is the area bounded by the middle third of the inguinal ligament, deep epigastric vessels, and a reach from the junction of the upper and middle thirds of the inguinal ligament, where the deep epigastric vessels cross posterior to the rectus abdominis muscle. TAPP, transabdominal preperitoneal

In cases of mesh repair for suprapubic incisional hernias that have a hernial orifice within 4 cm of the pubic tuberosity, the mesh should cover the hernial orifice and overlap it by 2-5 cm for fixation [7-10]. Thus, the Cooper ligament, pubic tuberosity, and, if necessary, the contralateral Cooper ligament should be exposed, and mesh should cover the hernial orifice sufficiently medially and ventrally to the hernia portal in supravesical hernias [7]. In addition, the inferomedial edge of the mesh should be fixed to the Cooper ligament/pubic tuberosity, as desufflation after TAPP repair tends to elevate the lower edge of the mesh and predisposes the inferomedial aspect to migrate from the Retzius space if a direct defect is present [11]. In summary, in TAPP repair of an external supravesical hernia, the mesh should cover an area of 2-5 cm around the hernial orifice, the Hesselbach triangle, and the lateral triangle, with the medial inferior margin securely anchored to the pubic tuberosity and Cooper ligament (Figure 7).

The use of 3D Light Mesh may be disadvantageous because of its small medial area; thus, a rectangular mesh may be preferable. This concept of the area of mesh repair is effective only for supravesical hernias protruding as inguinal and femoral hernias. In cases of obturator hernias, added coverage of the obturator foramen is necessary. It is unclear whether coverage of the Hesselbach and lateral triangles is necessary in patients with obturator hernias.

Ovarian prolapse in inguinal hernias is most commonly reported in girls, but it has also been reported in women [12-14]. In addition to inguinal hernias, femoral hernias [15,16], umbilical hernias [17], and Spigelian hernias [18] have been reported as forms of ovarian prolapse in women. A strangulated ovary can be released by an incision of the external inguinal ring and then placed in the abdominal cavity [14]. Irreducible ovarian hernia should be repaired as soon as possible [14]. In this case, although nine months had passed since onset, the ovary was preserved because there was no evidence of necrosis.

In our case, an external supravesical hernia coexisting with a hydrocele of the canal of Nuck, an indirect inguinal hernia, and a femoral hernia were not diagnosed preoperatively. Color Doppler ultrasonography is useful in the diagnosis of ovarian hernia and hernia-related complications, and it can be performed preoperatively [19]. Since hydrocelectomy and TAPP repair using mesh for hydroceles of the canal of Nuck have also been reported [20], TAPP repair may have been the first choice in our case for successful diagnosis by intra-abdominal observation.

## Conclusions

We encountered a case of an external supravesical hernia with an incarcerated ovary in a woman that was difficult to diagnose preoperatively. The laparoscopic approach was useful for the reliable diagnosis and reduction of incarcerated ovaries. Combining the concepts of mesh repair for direct and suprapubic incisional hernias, we believe that 2-5 cm around the hernial orifice, Hesselbach triangle, and lateral triangle should be covered with a mesh in the repair of external supravesical hernias. Further study is needed to confirm recurrence rates and postoperative complications associated with this procedure.
